# The Book World of Medicine and Science

**Published:** 1893-12-16

**Authors:** 


### THE BOOK WORLD OF MEDICINE AND SCIENCE.

### BOOK REVIEWS.

ON SEWAGE TREATMENT AND DISPOSAL. By THOMAS WARDLE. (Published by John Heywood. Pp. 408.)

The last word has not yet been said on sewage, nor is it likely to be for a good many years to come. In small towns and large institutions where natural conditions are at all favourable the problem of sewage disposal is not a very difficult one. It is even possible to solve it and remain solvent ourselves: perhaps even to make a profit; but when the sewage of a million-peopled city is in question it is extremely difficult; and in much smaller towns lying low in relation to the neighbouring soil which can be used for irrigation purposes we find ourselves surrounded by difficulties. Mr. Wardle's book will enable us to steer clear of some of these, and the care with which information has been obtained from all sources; the clear manner in which the various systems are described; the points in which one fails and the other succeeds so well brought out, that the volume will prove a most useful one not only to the inquirer, but to those whose duties compel them to make a selection of different methods for practical use.

The preface tells us that the book originally appeared as a series of newspaper articles, and we think it was a most desirable proceeding to link these articles in more permanent form. Chapter II. gives us the permissible impurities in the effluent water from sewage works allowed by the Thames Conservators' standard; that is, it should be free from offensive odour and suspended matters; not containing more than sixty grains of solids to the gallon. If reaction should not be distinctly acid or alkaline, it should not contain more than two grains of organic carbon to the gallon, and it should contain at least one cubic inch of free oxygen per gallon; and Dr. Buckland adds that effluents of even this high standard should not be discharged into the river if the water be afterwards used for domestic purposes. The third chapter is a good *résumé* of what is known on the bacteria question. The most of these micro-organisms are passed in review; authors quoted, and the various organisms are put before us in well executed drawings. We do not, however, notice any reference to Wynter Blyth's experiments on the anthrax bacillus.

In Chapter IV., which treats of the disposal of sewage, Robinson's table is reproduced, and the deductions from it are that 137 people require one acre at 38 gallons of sewage per day per individual. The value of sewage as manure is given at from a halfpenny to twopence per ton as a manure; but then it possesses a very high value as an irrigation agent merely, and we are inclined to think that twopence is much too low even as a manure.

One of the most useful chapters of reference in this book is that which gives a digest of the systems pursued in something like fifty-two of our English cities and towns. By glancing through this chapter, one can form a very fair idea as to which town further investigations ought to be pursued in. Of course the great difficulty in the treatment of sewage lies in rendering it pure enough to irrigate growing crops, and to enable the effluent to be subsequently discharged into some brook without nuisance or injury. Mr. Wardle does not

THE BOOK WORLD OF MEDICINE AND SCIENCE—*continued.*

believe in permitting crude sewage to be used on the land, and after detailing many plans of treating the crude article, he describes his own new method of precipitation, namely, that by basic persulphate of iron, ferricum, or ozonine.

We may add that the book is well printed and profusely illustrated, and is in many respects a most useful addition to the literature of the subject. At the risk of seeming a little hypercritical we refer to the profusion with which titles are sprinkled in the earlier pages of the volume. On the title page Mr. Wardle has thirteen different handles to his name, and in the preface Mr. Robinson is described as "H. Robinson, Esq., C.E., F.G.S., F.R.G.S." All this is, to say the least, unnecessary, and as the book is sure to be much read we hope it will be corrected in the next edition.

### DECEMBER REVIEWS.

An important contribution to the study of epidemic disease is furnished by M. A. Proust, of the Academy of Medicine, to the Revue des Deux Mondes. M. Proust has something to say concerning the spread of most of the great epidemics which have prevailed in ancient and modern times, his object being to prove that these diseases follow known routes, subject to the fluctuations of commerce and the improvements in methods of locomotion. The numerous well-disposed persons who invite their friends to come and amuse them as soon as they are smitten with influenza would do well to study M. Proust on the subject of the contagiousness of this malady. He lays special stress on the fact that the interval between an outbreak of influenza in St. Petersburg and its appearance in Paris has steadily decreased during the last 150 years in proportion as the means of communication have become more rapid, and that it leaps as a general rule from the capital first attacked to the farthest point on the line of communication (travelling as it were by express train) only to attack later the intermediate points. With regard to cholera M. Proust adduces many familiar instances to show the part played in the East by each successive new line of railway or extension of mercantile enterprise in carrying the disease to fresh regions or with increased speed to old points of attack. From the facts accumulated by numerous authorities it should be possible, he considers, to establish a system of international hygiene, and having regard to the uniform advance of epidemics by successive stages on well-known routes, to establish at given points sanitary stations destined to check the advance and protect us in the future. M. Proust is mysterious and gloomy in his opinion of English sanitation. It is "the situation acquired by England in Egypt which has created difficulties impossible to be ignored" in preserving Egypt and Europe from the cholera developed at Mecca, for "the sanitary doctrines of England are well-known, and what guarantee against the importation into Europe of exotic maladies of Eastern origin would remain," demands M. Proust, "if the sanitary administration of Egypt should become an English administration?" This ardent desire to flood Europe with cholera is exactly consistent with "the well-known sanitary doctrines" of perfidious Albion, but while Egypt has still left to her the bright example of Turkish hygiene, all is not lost.

